# Effect of Female Sex Hormones on the Immune Response against *Chlamydia abortus* and on Protection Conferred by an Inactivated Experimental Vaccine in a Mouse Model

**DOI:** 10.3390/pathogens11010093

**Published:** 2022-01-14

**Authors:** Laura Del Rio, Antonio Murcia-Belmonte, Antonio Julián Buendía, Jose Antonio Navarro, Nieves Ortega, Daniel Alvarez, Jesús Salinas, María Rosa Caro

**Affiliations:** 1 Departamento de Sanidad Animal, Facultad de Veterinaria, Regional Campus of International Excellence Campus Mare Nostrum, University of Murcia, 30100 Murcia, Spain; antonio.murcia1@um.es (A.M.-B.); nortega@um.es (N.O.); daniel.alvarez@um.es (D.A.); jsalinas@um.es (J.S.); mrcaro@um.es (M.R.C.); 2 Departamento de Anatomía y Anatomía Patológica Comparadas, Facultad de Veterinaria, Regional Campus of International Excellence Campus Mare Nostrum, University of Murcia, 30100 Murcia, Spain; abuendia@um.es (A.J.B.); jnavarro@um.es (J.A.N.)

**Keywords:** *Chlamydia abortus*, progesterone, estradiol, vaccine, mouse model, immune response

## Abstract

Mice are valuable models extensively used to test vaccine candidates against *Chlamydia abortus* and to clarify immunopathological mechanisms of the bacteria. As this pathogen has the ability to reactivate during pregnancy, it is important to deepen the knowledge and understanding of some of the effects of female hormones on immunity and vaccination. This study is aimed at describing the role of sex hormones in the pathology of OEA during chlamydial clearance using ovariectomised mice and also gaining an understanding of how 17β-oestradiol or progesterone may impact the effectiveness of vaccination. Animals were treated with sex hormones and infected with *C. abortus*, and the kinetics of infection and immune response were analysed by means of bacterial isolation, histopathology, and immunohistochemistry. In a second phase of the study, protection conferred by an experimental vaccine after hormone treatment was assessed. Oestradiol showed a stimulatory effect on the immune response during infection, with a more efficient recruitment of macrophages and T-cells at the infection site. Furthermore, after vaccination, oestradiol-treated animals showed a stronger protection against infection, indicating that this hormone has a positive effect, stimulating a specific memory response to the pathogen.

## 1. Introduction

*Chlamydia abortus* is the cause of ovine enzootic abortion (OEA). This pathogen with zoonotic potential is recognised as the main cause of reproductive failure in small ruminants and other domestic animals. This disease has a great economic impact in Europe and other parts of the world [1].

In common with other members of the Chlamydiaceae family, this obligatory intracellular bacterium follows a biphasic developmental cycle. In the sheep, infection is characterised by latency and reactivation during pregnancy manifested in the herd by sudden onset of abortions and stillbirths [2]. Several authors have studied the live cycle and pathogenesis of *C. abortus* in detail, contributing to a better understanding of mechanisms of transmission and prevention of OEA. In the natural host, the bacteria can infect non-pregnant ewes and produce latency, and once the ewe becomes pregnant, the infection reactivates and multiplies in the placental tissue, producing lesions and leading to late abortions or weak lambs ([3], reviewed in [4]). These foetal membranes and vaginal discharges can be a source of infection for both animals and humans [5,6].

Experimental infection with *C. abortus* in mice induces a clinical presentation that shares many similarities with the natural host, including late abortions and pneumonia, hence, murine models are considered well suited to study the immunopathological mechanisms of the disease, and consequently have been widely used to evaluate vaccine candidates (reviewed in [7]).

Latent infections followed by abortions at a certain time of gestation is the main characteristic of *C. abortus* infection, but the exact pathophysiological mechanisms that take place during the reproductive cycle accompanying these changes involving sex hormones are still, to a great extent, unknown. It has been shown that, in sheep, peripheral plasma levels of progesterone (P4) decreased during chlamydial infection, while local synthesis of 17β-oestradiol (E2) and prostaglandin in the tissue increased (reviewed in [8]).

Progesterone and oestradiol, have been described as the main reproductive female hormones to influence the progression of chlamydial infection both in vitro and in vivo. In particular, previous studies have suggested that susceptibility of epithelial cells of genital tract to chlamydial infection can be influenced by the hormonal status of the epithelium at the time of exposure. For example, in *C. trachomatis* infection, in vitro pre-exposure to progesterone and oestradiol significantly affected attachment of the bacteria to host cell membranes [9,10]. This could be explained by the fact that membrane oestrogen receptors may play a role in cell invasion and development of chlamydial inclusions, whereby the oestrogen environment may facilitate infection [10]. In female swine cells, it has been shown that susceptibility to cell infection by *C. suis* was higher when cells were collected during oestrogen-dominant phase of the oestrous than with cells obtained during progesterone-dominant phase [11].

Furthermore, in vitro studies have shown that sex hormone treatment could induce changes in the gene expression profile of chlamydia, specifically, up-regulation of genes compatible to a stress response, development of a persistence phenotype by oestradiol treatment and, by contrast, up-regulation of an energy efficiency gene response after progesterone exposure of infected cells [12]. Furthermore, sex hormones have been shown to induce phenotypical changes during *C. abortus* development in ovine cell lines in vitro. In the case of *C. abortus*, we have previously showed that not only was the infection rate increased in both endometrial and trophoblast cell lines after treatment with progesterone or oestrogen, but *C. abortus* aberrant forms were also induced by hormonal treatment in ovine endometrial cell line [13]. However, the persistent-like phenotype was not induced in the trophoblast cell line, suggesting that female sex hormones may affect the chlamydial development through changes in the host cell environment. Furthermore, in an in vitro *C. trachomatis* infection model, progesterone treatment was associated with a lower infectivity of cells, and it was related to an up-regulation of genes involved with innate immunity in these cells [14].

Sex hormones have also been reported to have an effect on immune responses. For example, it has been shown that variations in sex hormone levels can influence local immune responses to infections and this effect may vary between different parts of the female genital tract (reviewed in [15]). Furthermore, in a rodent model of intrauterine *C. trachomatis* infection, progesterone-treated animals showed increased susceptibility to infection through suppression of spleen-cell proliferation, while animals treated with oestradiol did not show this pathology [16].

Protection conferred after vaccination has also been shown to be altered in the same way by sex hormones in both human and mice studies. In a recent study of H1N1 vaccination, it has been reported that oestradiol treatment could benefit the production of vaccine-induced antibody responses in female mice [17]. In addition, a significant decline of antibody responses to herpes simplex virus has been reported in mice following vaccination after depot medroxyprogesterone acetate treatment (reviewed in [15]). In contrast, despite inducing a better protection in adult females than in males, depletion of oestradiol in female mice did not lower the level of protection conferred by a malaria vaccine in a mouse model [18].

Therefore, while the role of sex hormones on the immune system is well established, the knowledge about the specific role of oestradiol and progesterone during *C. abortus* infection and on protection conferred by vaccination remains scarce. Interestingly, our group has recently reported that, in an experimental *C. abortus* vaccination of sheep, progesterone treatment induced recruitment of Treg cells into inflammatory tissue, but did not affect either the humoral response or the cell-mediated immunity induced by immunisation [19]. To deepen the understanding of these effects, in the present study we have used an ovariectomised mouse model treated with oestradiol and progesterone to study the effect of sex hormones in the pathogenesis of *C. abortus* infection, and also their impact on the effectiveness of vaccination. This study provides further insight into the functioning of vaccine immunity under the effects of female hormones, which may help in the design of new vaccine strategies.

## 2. Results

### 2.1. Effect of Sex Hormone Treatment during C. abortus Infection

#### 2.1.1. Morbidity following Infection

**Figure 1:**
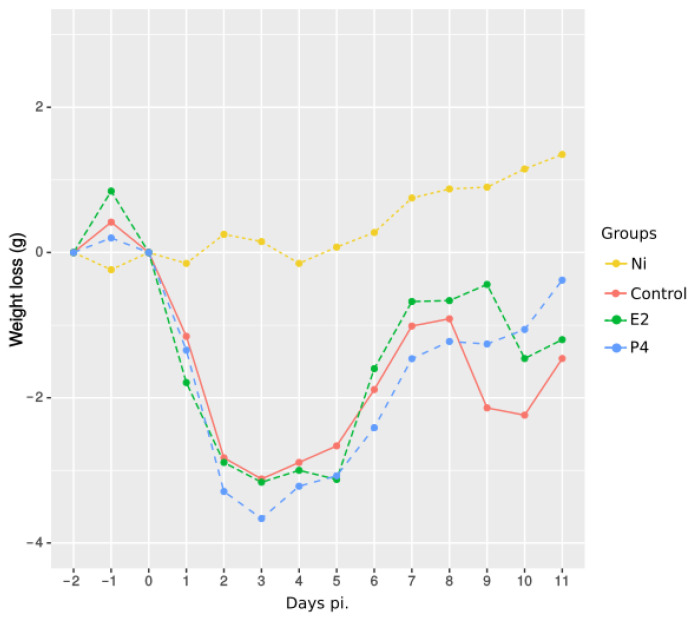
Morbidity following infection. Weight loss, expressed as mean of the change in weight of each individual mouse. Groups of mice (5 per group) were treated with oestradiol (E2) or progesterone (P4) and challenged with *C. abortus*. Non- treated-non-infected (Ni) and non-treated-infected (Control) groups were included in each experiment. Mann–Whitney-U-Test test was used to compare means between different days pi. and different treatments. All infected mice showed a significant decrease compared to Ni group from 1 to 11 days pi. Significant differences between non-treated-infected control and hormone treated groups were found only for E2 treated mice on day 9 pi. and for P4 treated mice at 10 and 11 days pi. (*p* < 0.05).

All infected mice experienced mild symptoms after *C. abortus* challenge, and morbidity was measured objectively by daily monitoring of body weight (Figure 1). Similarly, all infected mice started to lose weight 24 h after infection, reaching maximal weight loss between days 2 and 5 pi., followed by a fast recovery. This decrease was significant for all groups compared to non-infected mice from 1 to 11 days post infection, but by the end of the experiment, both E2- and P4-treated mice showed a better body condition than infected control mice, being significantly higher only for E2-treated mice on day 9 pi. and for P4-treated mice on days 10 and 11 pi.

#### 2.1.2. Bacterial Isolation

**Figure 2:**
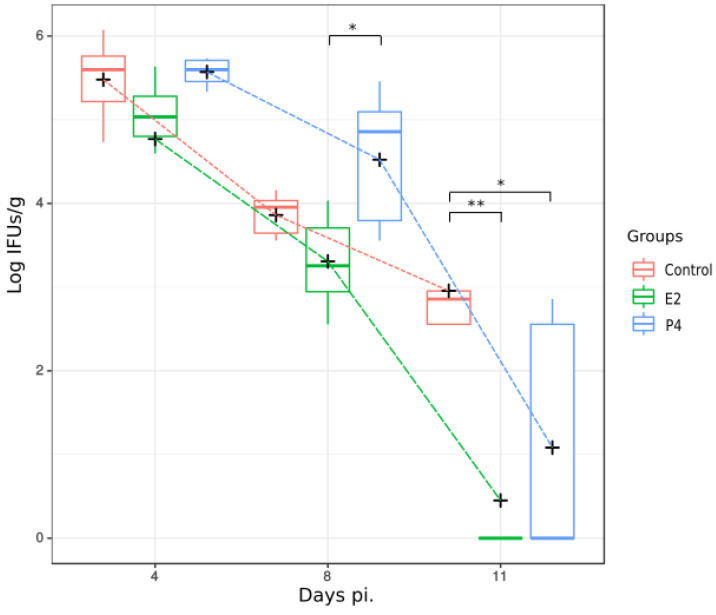
Level of *C. abortus* infection in the liver at different times after infection, evaluated as isolation of IFUs/g of liver tissue. Median (colour bars) and mean (black cross) for each group and time point are represented. ANOVA test was used to compare means between mouse groups and time points during the experiment. Although all groups of mice decreased the bacterial level, E2 hormone treatment group had a significant effect on clearing bacteria at 8 and 11 days pi, compared to P4 group and Control (* *p* < 0.05, ** *p* < 0.01).

*C. abortus* was isolated from the liver of infected mice, and the results were expressed as Log Inclusion Forming Units per gram (Log IFUs/g). Although the bacterial load decreased significantly on days 8 and 11 pi. in all groups, the E2-treated mice cleared the bacterial infection in the liver more efficiently and faster than the other groups. Thus, the amount of bacteria isolated from tissue of E2-treated mice on day 8 and 11pi was lower than other groups and close to 0 on day 11 (when 4 out of 5 mice had negative isolation), while the control group maintained higher levels of bacteria throughout the infection and the P4-treated mice still had some bacteria in their tissue on day 11 (when 2 out of 5 mice had negative isolation) (Figure 2).

#### 2.1.3. Recruitment of Inflammatory Cells to the Liver after Infection

Lesions in the liver were compatible with a multifocal granulomatous hepatitis in all groups. *C. abortus* antigen was also analysed in the liver by immunohistochemical labelling of LPS, where despite the fact that all groups showed a decreasing amount of antigen associated with inflammatory foci after day 8 pi., this decrease was more noticeable in the liver of E2-treated mice, while no inflammatory foci were present in non infected controls (data not shown).

**Figure 3:**
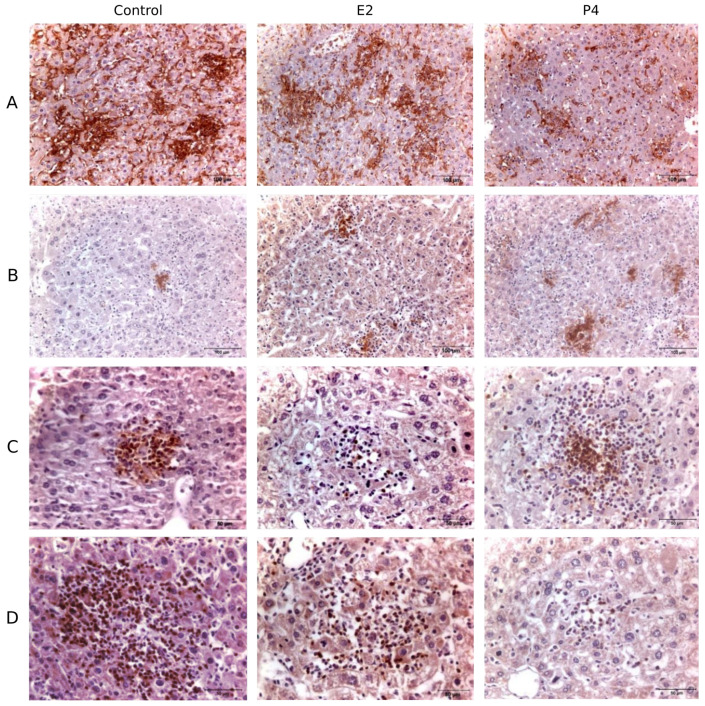
Characterization of the inflammatory infiltrate in the liver. Representative images of the hepatic lesions caused by *C. abortus* infection at day 8 pi. in non-treated infected control group (Control), oestradiol (E2) and progesterone (P4) treated mice. (**A**) Labelling with monoclonal antibodies against macrophages, (**B**) iNOS, (**C**) polymorphonuclear neutrophils (PMN), and (**D**) T lymphocytes (CD3).

**Figure 4:**
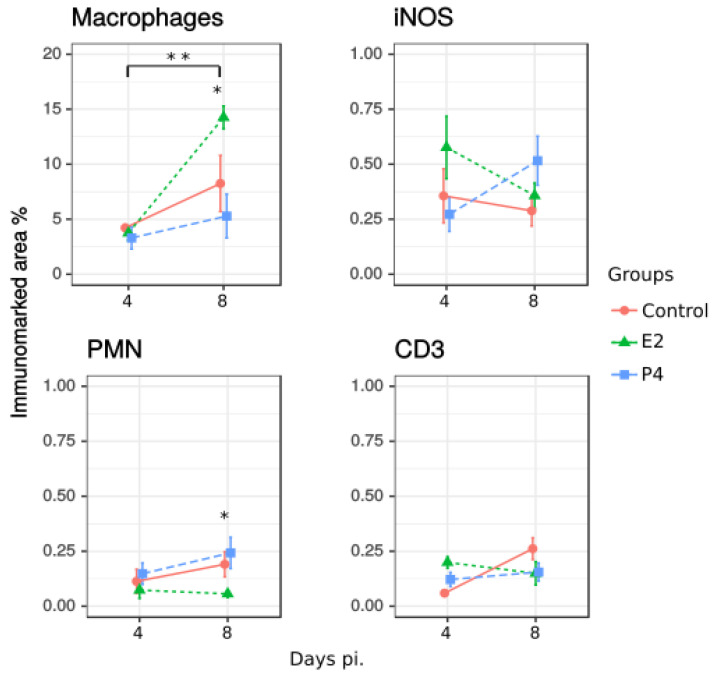
Characterization of the inflammatory infiltrate at different times of infection in non-treated infected control group (Control), oestradiol (E2) and progesterone (P4) treated mice, using monoclonal antibodies against macrophages, detection of iNOS-producing cells, polymorphonuclear neutrophils (PMN) and T lymphocytes (CD3). Quantification of each cell type was calculated as percentage of immunolabelled area. Results from ten different fields per mouse are shown. Significant differences between treatments and different days of infection were estimated by one-way-ANOVA. A significant increase was found in the recruitment of macrophages between 4 and 8 days pi. for all groups and the effect of E2 was significant on day 8 pi. The effect of E2 treatment was also significant in the recruitment of PMNs at 8 days pi. (* *p* < 0.05, ** *p* < 0.01).

To determine the composition of inflammatory foci and cell recruitment in the liver after chlamydial challenge, immunohistochemistry was used, with anti-F4/80 and iNOS to stain macrophages and iNOS-producing cells, respectively, and anti-NIMP-R14 and anti-CD3 to stain PMNs and T lymphocytes, respectively, (Figure 3). Then, percentage of immunolabelled areas per field was calculated and quantified for each group and marker, as described in Materials and Methods. An increase of immunolabelled area for macrophages from day 4 pi. to 8 pi. was significant in all infected groups, (Figure 4), furthermore E2-treated mice had significantly higher numbers of macrophage positive cells than the other groups at day 8pi. In addition, the recruitment of PMN was significantly lower in E2-treated mice than P4 mice on day 8 pi. (Figure 3 and Figure 4). However, when microbicide activity was determined by detection of iNOS, no effect of hormones on the number of iNOS-positive cells was detected, nor was there a statistically significant difference in the number of CD3-positive cells in tissue (Figure 4).

### 2.2. Effect of Hormone Treatment on Protection Conferred by the Vaccine

Protection conferred by the experimental inactivated vaccine was confirmed as immunised mice showed minor morbidity, a reduced bacterial load in the liver and produced specific antibodies.

#### 2.2.1. Morbidity in Vaccinated Mice after Treatment with Hormones

**Figure 5:**
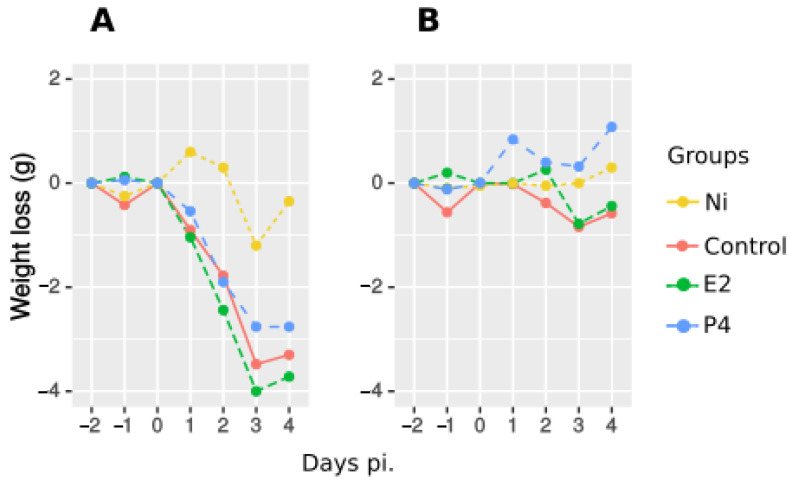
Morbidity following infection. Weight loss, expressed as mean of the change in weight of each individual mouse for non vaccinated mice (**A**) or vaccinated mice (**B**). Groups of mice were treated with E2 and P4 (days −2 −1 and 1 pi.) and challenged with *C. abortus*. Non treated non infected (Ni) and non treated-infected (Control) groups were included in each experiment. Mann–Whitney-U-Test test was used to compare means between different days pi. at each treatment. In non vaccinated mice, weight loss of hormone treated and infected control was similar until day 4 pi. All vaccinated mice kept their initial weight after infection, with no significant differences between groups.

Morbidity was calculated as weight loss (Figure 5). In non-vaccinated mice, hormone treated mice lost weight similarly to infected control mice from days 1 to 4 pi. (Figure 5A). By contrast, vaccinated mice did not show morbidity and there was no variability in weight loss between hormone-treated and control mice. (Figure 5B).

#### 2.2.2. Bacterial Isolation and Seroconversion after Vaccination

**Figure 6:**
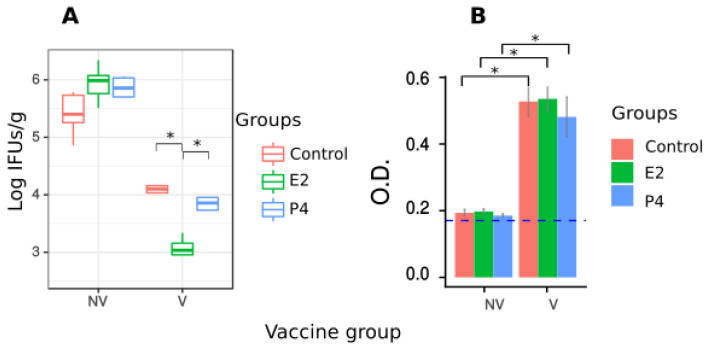
Effect of hormone treatment in vaccinated mice. (**A**) Level of *C. abortus* infection in the liver of vaccinated and non vaccinated mice, evaluated as isolation of IFUs/g of liver tissue at 4 days pi. Distribution of data for each mouse group and treatment is represented. Vaccinated groups had a significantly lower level of bacteria than non vaccinated for each treatment. E2-treated group showed the best protection after vaccination as level of bacteria was significantly lower than both control and progesterone treated groups. (**B**) Specific antibody level in sera measured by ELISA at 4 days pi. Blue dotted line represents the level of antibodies from sera of non infected non vaccinated mice. Antibody titer increases significantly in all groups tested after vaccination. ANOVA test was used to compare the effect of vaccination and different hormone treatments. (* *p* < 0.05).

The effect of hormone treatment after vaccination was also studied by analysing the bacterial clearance in the liver and production of anti-*C. abortus* specific antibodies in vaccinated mice at 4 days pi. (Figure 6). All vaccinated mice were able to succesfully clear bacteria and they presented a significantly lower number of IFUs/g compared to non-vaccinated mice. However, when comparing the effect of different hormone treatments in bacterial clearance, only in E2-treated vaccinated mice was there a significantly lower number of IFUs/g than in the control group, while progesterone did not have this effect (Figure 6A). In addition, all hormone-treated and non-treated mice showed seroconversion with similar levels of anti-*C. abortus* antibodies in sera following immunization with the experimental vaccine (Figure 6B).

#### 2.2.3. Immunohistopathology

**Figure 7:**
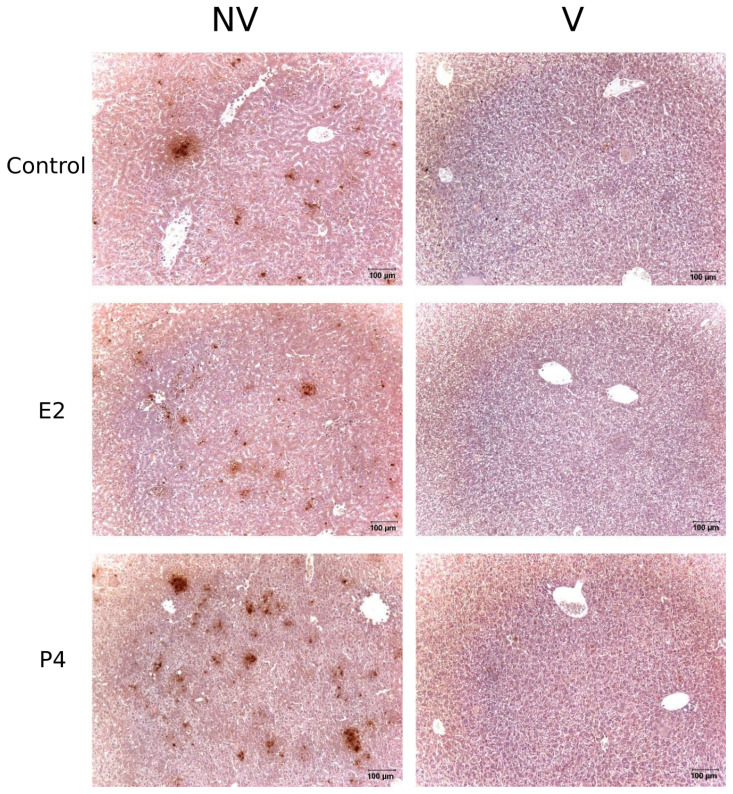
Detection of *C. abortus* antigen in the liver of non-treated infected control group (Control), oestradiol (E2) and progesterone (P4) treated mice in non vaccinated (NV) and vaccinated mice (V) at 4 days after infection. Vaccinated mice showed a significant decrease of positive staining compared to non vaccinated mice and this effect is consistent in hormone treated groups.

When the protective effect of vaccination was studied by immunohistochemistry in the liver, there was a reduction in the numbers of foci of cellular infiltrate associated with positive staining of chlamydial antigen in all groups, (Figure 7); this reduction was similar in all groups and no significant effect of the hormone treatment could be detected for neither E2 or P4 treated mice. These results suggest that the protection conferred by the experimental vaccine was not affected by the hormone treatment.

#### 2.2.4. Effect of Hormone Treatment on Cell Recruitment after Vaccination

**Figure 8:**
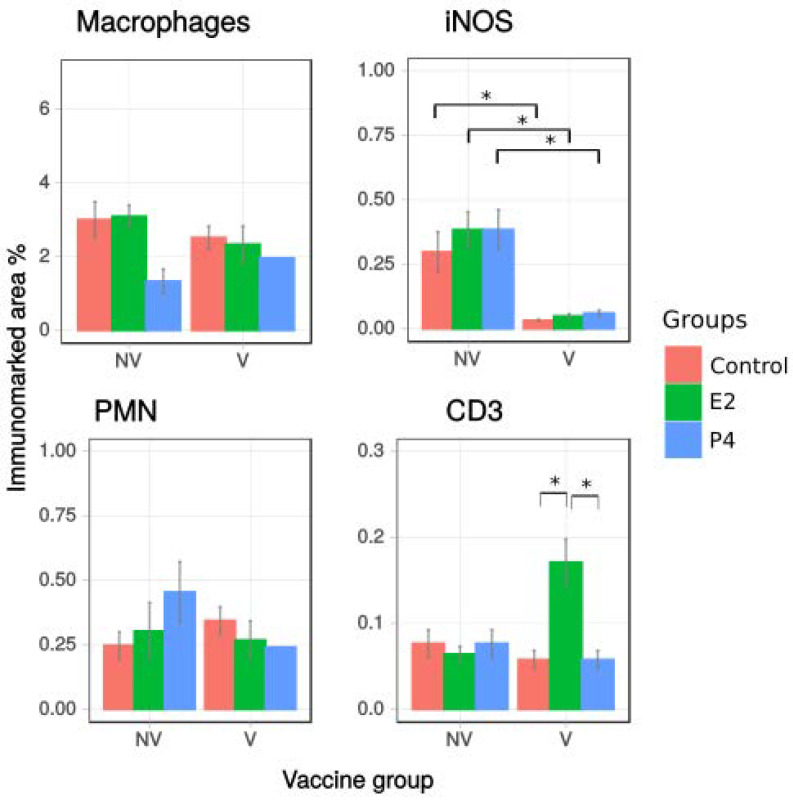
Characterisation of the inflammatory infiltrate in non-treated infected control group (Control), oestradiol (E2) and progesterone (P4) treated mice in non vaccinated (NV) and vaccinated mice (V). Monoclonal antibodies against macrophages, detection of iNOS from iNOS-producing cells, polymorphonuclear neutrophils (PMN) and T lymphocytes (CD3) were used. Quantification of each cell type was calculated as percentage of immunolabelled area. Results from ten different fields per mouse are shown. Significant differences between treatments and vaccination were estimated by one-way-ANOVA. iNOS labelling was significantly lower in all vaccinated mice than non-vaccinated groups. E2 treatment had a significant effect in the recruitment of T lymphocytes with higher percentage of positive area than Control and P4 groups (* *p* < 0.05).

Immunohistochemistry analysis was performed with differential staining for different cell types in order to quantify the presence of these cell types in the tissue of vaccinated mice at day 4 post challenge (Figure 8). In all groups of vaccinated mice the proportion of iNOS-positive cells was significantly lower compared to the non-vaccinated mice. However, an effect of vaccination was not detected in the recruitment of macrophages and PMNs. On the other hand, the effect of hormone treatment was significant only for T lymphocyte recruitment. Specifically, in vaccinated mice, there was a higher percentage of CD3 immunolabelled area in E2-treated mice than in control mice and P4-treated mice.

## 3. Discussion

The results of the present study have shown that oestradiol treatment induces changes in the outcome of *C. abortus* infection, leading to a shorter duration of the infection period, with lower bacterial burden than in progesterone-treated and infected control groups. These changes were accompanied by increased macrophage recruitment.

Oestrogen has been shown to enhance immune responses to several infectious agents ([20]). Specifically, the relationship of oestradiol treatment and increased iNOS has been shown previously ([21]). It should be noted that sex hormones not only control the local and systemic immune response but also modulate cell-signalling gene expression in potential host cells. For example, oestrogens have also been shown to regulate the transcription factors Stat-1 and NF-κβ for iNOS production and to increase the inflammatory response in mouse splenocytes ([22]). In another study, oestradiol have been described to modulate CD4 T-lymphocyte activity by increasing their proliferation and cytokine production, as well as enhancing their antigen-specific binding efficiency ([23,24]). In addition, in our study the increased efficacy in eradicating chlamydial infection in oestradiol-treated mice was accompanied by significant elevation of macrophages at inflammatory foci in the late phase of infection (8 days pi.). This is in accordance with other studies where the role of oestradiol as a regulator of cell recruitment to sites of inflammation, diversifying cell populations and elevating the number of macrophages relative to neutrophils, was established ([25]). It has also been shown that oestradiol is able to activate macrophages to increase their production of Th1-activating cytokines ([26]), a response necessary for the elimination of chlamydial infection. It has been reported that, in the natural host as well as in murine experimental models, an effective immune cell response is crucial for the elimination of *C. abortus* infection (reviewed in [7]), these mechanisms could explain the more efficient resolution of chlamydial infection in oestradiol-treated mice.

The complex relationship between sex hormone production and infection with *Chlamydia* spp. has recently been reviewed by [27]. Analysis of infection with *C. trachomatis* in animal models have shown that there are significant differences in the effect of the sex hormone on immunity, depending on the host species. For example, some authors have shown that progesterone pre-treatment facilitated chlamydial multiplication and dissemination in mice ([28]), but another study using guinea pigs as hosts showed that, it was the oestradiol pre-treatment that produced longer infections, with greater dissemination and pathology ([29]). In the case of *C. abortus*, a previous study of experimental vaccination in the natural host, progesterone was shown to have a role enhancing a protective immune response after immunisation with the same experimental vaccine used in this study ([19]). However, in the present study we have described that, in mice, progesterone treatment exerted no positive or detrimental effect on the development of protective response induced by vaccination. This apparent variance in the role of progesterone during vaccination could be explained by differences in the animal model.

On the other hand, our results show that oestradiol treatment could enhance the protection conferred by the vaccine in the mouse. Specifically, in oestradiol-treated animals, a lower infectious burden in the liver and an increased number of T-cells in the inflammatory infiltrate of the lesions were observed. This result is consistent with other authors, as the treatment with oestradiol has been shown to improve efficacy of vaccination against various intracellular pathogens in several animal models. For example, oestradiol has been described to enhance the neutralising antibody titre in mouse models of infection with genital herpesvirus type II (HSV-2) infection ([30,31]) and H1N1 [17]. Furthermore, in another vaccination model of HSV-2, oestradiol has been shown to increase dendritic cell activity by producing inflammatory cytokines such as IL-1beta [32] and more recently, this hormone has also been shown to increase the number of CD4+ memory T cells in tissue through a IL-17 mediated pathway [33].

In summary, the present study shows that oestradiol treatment was associated with a faster resolution of the infection, related to a higher recruitment of macrophages and T cells in vaccinated mice, while progesterone treatment did not have any positive nor detrimental effect in relation to the infection in these mice. These results suggest that oestradiol, and not progesterone, may have an effect on the specific memory response in vaccinated mice. This study provides further insight into the effects of female hormones on the functioning of vaccine immunity, which in the case of abortion, may be useful for development of new vaccine strategies.

## 4. Materials and Methods

### 4.1. Ethics Statement

Female mice of Swiss OF1 background, (Janvier Lab) were kept in a specific pathogen-free facility at the University of Murcia. This study was carried out in accordance with the principles of the Basel Declaration and recommendations of the Spanish Government (Comunidad Autónoma de la Región de Murcia) concerning protection of the animals for experimentation. The experimental design was approved by the Committee on the Ethics of Research in Animal Experimentation of the University of Murcia, Spain. Protocol number: A13150203.

### 4.2. Microorganisms

*C. abortus* (AB7 strain) was propagated in the yolk sacs of developing chick embryos and titrated by counting inclusion-forming units (IFUs) in McCoy cells, as previously described [34]. Standardized aliquots were frozen at −80 °C until use.

### 4.3. Experimental Design

**Figure 9:**
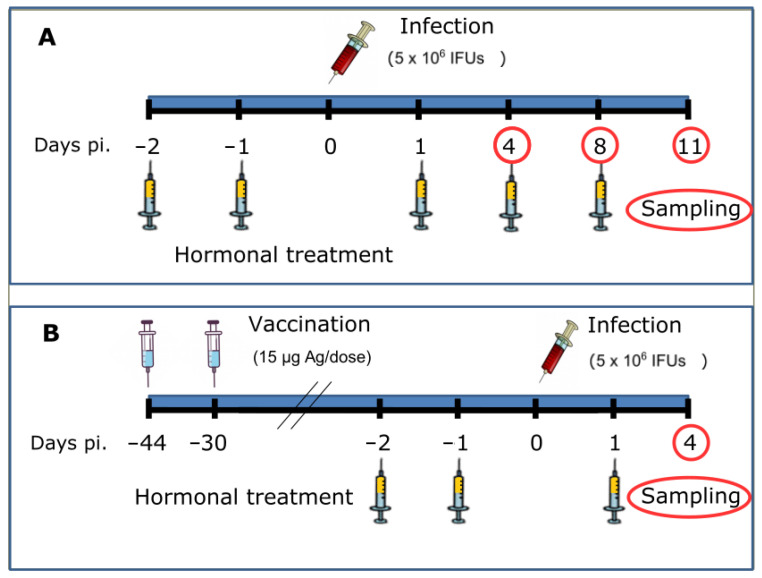
Experimental designs. (**A**) Phase 1. Mice were treated with either E2 or P4 at 2 days and 1 day before challenge and at 1, 4 and 8 days pi. Infection dose was $5\times 10^{6}$ IFUs at day 0 and groups of 5 animals were killed to take samples of blood and liver at 4, 8 and 11 days pi. (**B**) Phase 2. Experimental vaccination was done in two doses 44 and 30 days before challenge. Then, hormone treatment was done 2 and 1 days before challenge and 1 day pi. All mice were killed and sampled 4 days pi.

This study was divided into two phases. In the first stage, the model of hormone treatment and infection was established. The bacterial challenge was recorded as day 0. In the second stage, the effect of hormone treatment during the development of the immune response after infection was studied in vaccinated mice. All the experiments were repeated twice for reproducibility. (Figure 9).

#### 4.3.1. Phase 1. Hormone Treatment and Infection of Mice

Mice of six weeks of age underwent bilateral ovariectomy following the methodology previously described [35]. Then, animals were distributed into groups of 5 and treated subcutaneously on days −2, −1, 1, 4, and 8 post infection (pi.) with either oestradiol (E2) (3 μg in 0.2 mL of sesame oil/mouse), or progesterone (P4) (3 mg in 0.2 mL of sesame oil/mouse). Non-infected (Ni) and infected control (Control) mice were injected 0.2 mL of sesame oil without hormones (Figure 9A). For experimental infection, mice were challenged intraperitoneally with $5\times 10^{6}$ IFUs of *C. abortus* AB7 strain in 0.2 mL of 0.1 M phosphate-buffered saline (PBS), pH 7.2 and their clinical status was recorded daily. Non-infected mice (5 animals) were injected 0.2 mL of sterile PBS. Mice were killed on days 4, 8, and 11 pi. (Figure 9A). Blood was collected by cardiac puncture, and liver samples were collected and stored at −80 °C until bacteriological analysis. In addition, tissue samples from liver were fixed in 10% formalin or Zinc fixative agent (BD Biosciences, Pharmingen, San Jose, CA, USA) and embedded in paraffin for further histopathological or immunohistochemical studies, respectively.

#### 4.3.2. Phase 2. Vaccination, Hormone Treatment and Infection of Mice

Mice were vaccinated with an experimental inactivated vaccine previously tested and validated in our laboratory using mouse models [7] and ovine models [36,37]) using a purified derivate of saponin (QS21; Agenus) as adjuvant. The vaccine was prepared according to the protocol described by [38]. Vaccinated mice received two doses of the vaccine subcutaneously (15 μg of *C. abortus* proteins in 0.2 mL), 43 and 29 days before the infection. Then, mice were distributed in groups of 5 and treated subcutaneously on days −2, −1, and 1 pi. with either E2 or P4 as previously described in Section 4.3.1. Then, mice were challenged intraperitoneally with *C. abortus* AB7 strain, killed on day 4 pi. and blood and liver samples were collected as previously described in Section 4.3.1. (Figure 9B).

### 4.4. Morbidity and Course of Infection

Mice were monitored daily for possible signs of infection and morbidity was determined as mean of individual weight loss per day for each group. In addition, the course of *C. abortus* infection in mice was evaluated as bacterial clearance in liver tissue. This organ has previously been described as the target organ after intraperitoneal infection with similar infection kinetics than the spleen [39]. The level of infection was measured by titration of IFUs per grams of tissue on monolayers of McCoy cells, as described in [34].

### 4.5. Detection of Anti-C. abortus Antibodies in Sera

To monitor the protective response to the vaccine, serum samples were analysed for the production of *C. abortus*-specific antibody levels by commercial ELISA (ID Screen *Chlamydophila abortus* indirect multispecies, IDvet) following the instructions of the manufacturer. In addition, specific anti-mouse IgG (Sigma, Madrid, Spain) was used as the conjugate for the reaction, and positive and negative control mouse sera were added in order to increase specificity.

### 4.6. Histopathology and Immunohistochemistry

Formalin-fixed liver sections (4 μm) were stained with haematoxylin-eosin staining for histopathological study and immunohistochemical labelling was carried out to demonstrate presence of chlamydial antigen (Abcam, Cambridge, UK) as previously described [34]. Additionally, characterisation of the inflammatory infiltrate was carried out in Zinc-fixed samples, using monoclonal antibodies against F4/80 for macrophages (Caltag Medsystem, Buckingham, UK), iNOS (Upstate Biotechnology, Lake Placid, NY, USA) that can be present in activated macrophages and other iNOS-producing cells, reviewd in [40], CD3 for T lymphocytes (Dako-Agilent, Santa Clara, CA, USA), and NIMP-R14 for polymorphonuclear neutrophils (PMN) (Santa Cruz Biotechnology, Dallas, TX, USA), using Avidin-Biotin-Peroxidase Complex (ABC) (as described in [41]). Isotype control antibodies were used to assess the non-specific background staining. To quantify the distinct cell types, the stained area was analysed for each marker in ten fields per sample, quantified and expressed as percentage of stained area per field, as previously described [42].

### 4.7. Statistical Analysis

To compare means of two independent samples (vaccinated and non-vaccinated or hormone-treated groups and Controls), a non-parametric Mann–Whitney U test was used. A one-way ANOVA test was used to compare means between hormone treated and control groups, and changes in inflammatory infiltrate over time with the percentage of stained area as the measurement variable and sex hormone treatment and time after infection or vaccination as the factors. As post hoc test, Tukey’s multiple comparisons of means with 95% confidence level was used. All analyses were performed using R (R version 3.4.4) [43] and criteria for reproducible research were followed ([44]). A *p* value of <0.05 was considered statistically significant.

## 5. Conclusions

Under the experimental conditions established in this study, oestradiol promotes the resolution of a *C. abortus* infection, while progesterone does not interfere significantly in the resolution of the infection or in the triggered immune response.

Oestradiol, administered before and after challenge in vaccinated mice, enhances the conferred protection of an experimental inactivated vaccine against *C. abortus*, associated with increased recruitment of T lymphocytes to the target tissue.

## Data Availability

Not applicable.

## Abbreviations

The following abbreviations are used in this manuscript:

OEA	Ovine enzootic abortion
E2	Oestradiol
P4	Progesterone
LPS	Bacterial lipopolysaccharides
PMNs	Polymorphonuclear neutrophils
IFUs	Inclusion forming units
iNOS	Inducible nitric oxide synthase
ELISA	Enzyme-Linked ImmunoSorbent Assay
